# Andrographolide prevents necroptosis by suppressing the generation of reactive oxygen species: Andro prevents necroptosis by suppressing the generation of ROS

**DOI:** 10.3724/abbs.2025077

**Published:** 2025-05-28

**Authors:** Na Lu, Qing Li, Linghan Duan, Rong Xu, Yaping Li, Fuli Shi, Zhiya Zhou, Yingqing Gan, Bo Hu, Jinhua Li, Xianhui He, Dongyun Ouyang, Qingbing Zha

**Affiliations:** 1 Guangdong Provincial Key Laboratory of Spine and Spinal Cord Reconstruction the Fifth Affiliated Hospital (Heyuan Shenhe People’s Hospital) Jinan University Heyuan 517000 China; 2 Department of Immunology and Microbiology College of Life Science and Technology Jinan University Guangzhou 510632 China; 3 State Key Laboratory of Bioactive Molecules and Druggability Assessment Jinan University Guangzhou 510632 China; 4 Center of Reproductive Medicine the First Affiliated Hospital of Jinan University Guangzhou 510632 China; 5 Department of Nephrology the First Affiliated Hospital of Jinan University Guangzhou 510632 China; 6 Department of Ultrasound the Fifth Affiliated Hospital of Jinan University (Heyuan Shenhe People’s Hospital) Heyuan 517000 China; 7 Department of Ultrasound the First Affiliated Hospital of Jinan University Guangzhou 510632 China

**Keywords:** andrographolide, necroptosis, mitochondria, reactive oxygen species, necrosome, Nrf2

## Abstract

Andrographolide (Andro), a natural product extracted from the Chinese traditional medicine herb *Andrographis paniculata*, has been applied for the treatment of diverse inflammatory diseases. However, its effects on necroptosis, a lytic form of cell death implicated in various inflammatory diseases, remain uncharacterized. In the present study, we investigate whether Andro and its derivatives can suppress necroptosis. Our results demonstrate that Andro notably inhibits necroptosis in the *in vitro* cellular models induced by either lipopolysaccharide (LPS) plus IDN-6556 or a combination of TNF-α, LCL-161 (Smac mimetic) and IDN-6556. In these cellular models, Andro inhibits the phosphorylation of receptor-interacting protein kinase 1 (RIPK1), RIPK3, and mixed lineage kinase domain-like pseudokinase (MLKL), as well as the formation of necrosomes. Specifically, Andro reduces the levels of intracellular reactive oxygen species (ROS) and mitochondrial superoxide (mtROS), preserves the mitochondrial membrane potential during necroptotic induction, and activates the antioxidant transcription factor nuclear factor E2-related factor 2 (Nrf2). Upon necroptotic stimulation, some mitochondrial proteins, such as Bcl-2 and Bak, oligomerize and co-localize with RIPK1, RIPK3, and phosphorylated MLKL (p-MLKL) in necrosomes. However, this process of necrosome formation can be prevented by Andro. In contrast, derivatives, including dehydroandrographolide, neoandrographolide, 14-deoxy-11,12-didehydroandrographolide, and 14-deoxyandrographolide, have no anti-necroptotic effects and fail to upregulate Nrf2. Collectively, our findings demonstrate that Andro specifically inhibits the RIPK1/RIPK3/MLKL signaling axis to suppress necroptosis, highlighting its therapeutic potential against necroptosis-related disorders.

## Introduction

*Andrographis paniculata*, a Chinese traditional medicine, is widely used to treat inflammatory diseases, including gastroenteritis, tracheitis, lung abscess, cholecystitis, and oropharyngeal swelling/pain. Andrographolide (Andro), a diterpene lactone compound isolated from this plant, has demonstrated therapeutic efficacy against a number of inflammatory conditions, such as pneumonia [1], colitis [ 2, 3], myocarditis [4], and rheumatoid arthritis [5].

Previous studies have revealed multiple mechanisms underlying the anti-inflammatory effects of Andro. First, Andro covalently modifies cysteine residues within the nuclear factor (NF)-κB p50 subunit to suppress NF-κB signaling [ 6, 7]. Second, Andro can activate nuclear factor E2-related factor 2 (Nrf2) [8]. Third, Andro can inhibit inflammasome activation, thus preventing pyroptosis [ 9, 10]. Despite these advances, more investigations are warranted for a comprehensive understanding of its anti-inflammatory mechanisms.

Regulated cell death (RCD) is a key event in inflammatory organ injury. Among the various forms of RCD, necroptosis critically influences the modulation of physiological and pathological processes, causing a range of human diseases, including ischemic brain injury, immune system diseases, and cancer [11]. This lytic cell death is characterized by disruption of the cell membrane, resulting in the release of cellular components and inflammatory mediators, consequently triggering inflammation. Unlike other forms of RCD, necroptosis is independent of cysteinyl aspartate-specific proteinases (caspases) but is controlled by the receptor-interacting protein kinase 1 (RIPK1)/RIPK3/mixed lineage kinase domain-like pseudokinase (MLKL) signaling pathway. Mechanistically, phosphorylated RIPK1 recruits and phosphorylates RIPK3 [ 12, 13], thus forming a necrosome complex that subsequently phosphorylates/activates MLKL. Activated MLKL translocates to the plasma membrane and oligomerizes to form membrane-disrupting pores [14], which can be inhibited by necrostatin-1 (Nec-1), a specific RIPK1 inhibitor [15]. This necroptotic signaling pathway can be initiated by the binding of tumor necrosis factor (TNF) to its receptor TNFR1, viral nucleic acids, *etc*. [ 16, 17]. Therefore, targeting necroptosis appears to be an effective and feasible approach for treating such diseases.

In the present study, we investigated the potential of Andro to inhibit necroptosis as a novel anti-inflammatory mechanism. Our evidence revealed that Andro effectively attenuates the necroptosis induced by TNF-α/LCL-161 (Smac mimetic)/IDN-6556 (a pan-caspase inhibitor) or LPS/IDN-6556. Mechanistically, Andro inhibits the activation of the RIPK1-RIPK3-MLKL signaling pathway but decreases intracellular reactive oxygen species (ROS) accumulation and mitochondrial superoxide (mtROS) production, thereby preserving the mitochondrial membrane potential (MMP). Notably, four structural derivatives of Andro lack comparable inhibitory activities. *In vivo*, Andro alleviates the uterine injury induced by TNF-α in C57BL/6J mice. These findings suggest that Andro is a promising therapeutic candidate for necroptosis-mediated inflammatory diseases.

## Materials and Methods

### Reagents and antibodies

Andrographolide (A823169) was purchased from Macklin (Shanghai, China) and dissolved in dimethyl sulfoxide (DMSO). 14-deoxy-11,12-didehydroandrographolide (B30518), 14-deoxyandrographolide (B24387), neoandrographolide (B21392), and dehydroandrographolide (B21275) were purchased from Yuanye (Shanghai, China). Lipopolysaccharide (LPS, *Escherichia coli* O111:B4), Hoechst 33342, propidium iodide (PI), DMSO, and DL-dithiothreitol (DTT) were purchased from Sigma-Aldrich (St Louis, USA). IDN-6556 (S7775) and LCL-161 (S7009) were purchased from Selleck Chemicals (Houston, USA). Fetal bovine serum (FBS) and Dulbecco’s modified Eagle’s medium (DMEM) supplemented with high glucose, streptomycin, and penicillin were purchased from Thermo Fisher Scientific (Carlsbad, USA). Specific antibodies against β-actin (#3700), phospho(p)-RIPK1 (#53286), p-RIPK3 (#91702), p-MLKL (#37333), RIPK1 (#3493), RIPK3 (#95702), MLKL (#37705), horseradish peroxidase (HRP)-conjugated horse-anti-mouse IgG (#7076), and HRP-conjugated goat-anti-rabbit IgG (#7074) were purchased from Cell Signaling Technology (Danvers, USA). Murine TNF-α (315-01A) was purchased from PeproTech (Rocky Hill, USA). Human TNF-α (ab9642) was obtained from Abcam (Cambridge, UK).

### Cell culture

The murine macrophage line J774A.1 was obtained from Kunming Cell Bank, CAS (Kunming, China), and cultured in complete DMEM supplemented with 10% FBS, 100 μg/mL streptomycin, and 100 U/mL penicillin. For the experiments, J774A.1 cells were seeded at a density of 8 × 10 ^4^ cells/well in a 24-well plate or 3.2 × 10 ^5^ cells/well in a 6-well plate. Human HT-29 colorectal adenocarcinoma cells were obtained from the Cell Bank of the Chinese Academy of Sciences (Shanghai, China) and cultured in complete McCoy’s 5A medium (Merck, San Diego, USA). For the experiments, HT-29 cells were seeded at a density of 1.4 × 10 ^5^ cells/well in a 24-well plate.

Bone marrow-derived macrophages (BMDMs) were obtained as previously reported [18]. Briefly, bone marrow cells were harvested from the hind femora and tibias of the mice and seeded in a 10-cm petri dish containing 10 mL of BM-Mac medium (containing 80% complete DMEM and 20% M-CSF-conditioned medium derived from L-929 cells). After 3 days, each dish was supplemented with 5 mL of BM-Mac medium, and the cells were cultured for another 3 days. For the experiments, BMDMs were seeded in a 24-well plate (2.5 × 10 ^5^ cells/well) or a 6-well plate (1.6 × 10 ^6^ cells/well). All the cells were incubated in a humidified incubator with 5% CO _2_ at 37°C.

### Induction of necroptosis

Necroptosis was induced as reported previously [19]. A combination of TNF-α (T, 30 ng/mL), LCL-161 (S, 10 μM for BMDMs and J774A.1 cells or 50 μM for HT-29 cells), IDN-6556 (I, 30 μM) (hereafter referred to as TSI), or a combination of LPS (L, 0.5 μg/mL) and IDN-6556 (I, 30 μM) (hereafter referred to as LI) was used for the indicated durations.

### Cell death assay

Cell death was evaluated by a PI incorporation assay as previously described [20]. After the indicated treatments, the cells were stained with a solution containing 2 μg/mL PI and 5 μg/mL Hoechst 33342 in sterile PBS in the dark for 10 min. Then, the cells were observed under a Zeiss Axio Observer D1 microscope (Zeiss, Gottingen, Germany), and images were captured.

### Western blot analysis

Western blot analysis was performed as previously described [21]. Proteins in the cell lysates were separated by sodium dodecyl sulfate-polyacrylamide gel (SDS-PAGE) electrophoresis and subsequently transferred to PVDF membranes (03010040001; Roche, Mannheim, Germany). The membranes were then blocked with blocking buffer for 1 h. After that, the membranes were incubated with the indicated antibodies at 4°C overnight. The membranes were subsequently incubated with a suitable HRP-conjugated antibody and then treated with an enhanced chemiluminescence kit (BeyoECL Plus; Beyotime, Shanghai, China) to visualize the target bands, which were then captured on X-ray films (Carestream, Xiamen, China).

### Immunofluorescence microscopy

Immunofluorescence (IF) analysis was performed as previously described [22]. Briefly, BMDMs were seeded in glass-bottom dishes (2.5 × 10 ^5^ cells). After the indicated treatments, the cells were fixed with 4% paraformaldehyde in PBS for 15 min, followed by permeabilization in cold methanol at –20°C for 10 min. Next, the cells were blocked with a blocking solution (5% goat serum in PBS) for 1 h and then incubated with the indicated antibodies (1:300) at 4°C overnight. After that, the cells were stained with CF568-conjugated goat-anti-rabbit IgG (#SAB4600084; Sigma-Aldrich) (1:300) and CF488-conjugated goat-anti-mouse IgG (#SAB4600237; Sigma-Aldrich) (1:300) for 1 h. Subsequently, the cells were stained in PBS solution containing Hoechst 33342 for 10 min. Cell images were captured using a Zeiss Axio Observer D1 microscope.

### Detection of intracellular ROS and mitochondrial superoxide

As previously described [23], an ROS assay kit (S0033; Beyotime) was used to evaluate intracellular ROS levels, whereas MitoSOX Red (M36008; Thermo Fisher Scientific) was used to measure the levels of mitochondrial superoxide (mtROS). After the indicated treatments, the cells were assayed with the above reagents in an incubator for the appropriate durations following the instructions of the suppliers. Finally, the fluorescence of DCFH-DA or MitoSOX within the cells was examined using a Zeiss Axio Observer D1 microscope.

### Detection of the MMP

The MMP was determined using a TMRE assay kit (C2001; Beyotime) and a JC-1 probe (C2006; Beyotime). After the indicated treatments, the cells were incubated with 1 μg/mL TMRE or 5 μg/mL JC-1 staining solution following the instructions of the suppliers. The intracellular red fluorescence of TMRE was observed using a Zeiss Axio Observer D1 microscope, and JC-1 staining was analyzed using a flow cytometer (Attune NxT; Thermo Fisher Scientific).

### TNF-α-induced systemic inflammatory response syndrome (SIRS) model

C57BL/6J mice (6–8 weeks of age) purchased from the Guangzhou Ruige Biological Technology Company (Guangzhou, China) were randomly assigned to 4 groups ( *n* = 5 per group): the vehicle group, Andro group, TNF-α group, and Andro + TNF-α group. The mice in the TNF-α and Andro + TNF-α groups were intravenously ( *i*. *v*.) injected with a single dose of TNF-α (5 μg per mouse), whereas those in the vehicle group were intravenously injected with sterile PBS. The mice in the Andro and Andro + TNF-α groups were intraperitoneally ( *i*. *p*.) administered with Andro (0.5 mg/kg or 1 mg/kg body weight) three times (1 h before and 2 h and 5 h after TNF-α injection, respectively). Nine hours after TNF-α administration, the mice were anaesthetized and sacrificed by cervical dislocation, after which uterus tissues were collected.

### Histological staining

Collected uterus tissues were fixed in 4% paraformaldehyde for 24 h and then embedded in paraffin. The paraffin sections of the uteri in 5 μm thickness were routinely prepared and stained with hematoxylin and eosin (H&E) and observed under a Zeiss Axio Observer D1 microscope.

### Statistical analysis

The data are presented as the mean ± standard deviation (SD) and were analyzed for statistical significance using GraphPad Prism 7.0 software (GraphPad Software Inc., San Diego, USA). The statistical significance among multiple groups was determined via one-way analysis of variance (ANOVA) followed by Tukey’s post hoc test. Statistical significance was defined as *P* < 0.05.

## Results

### Andro inhibits TSI- or LI-induced necroptosis in macrophages and HT-29 cells

**Figure 1:**
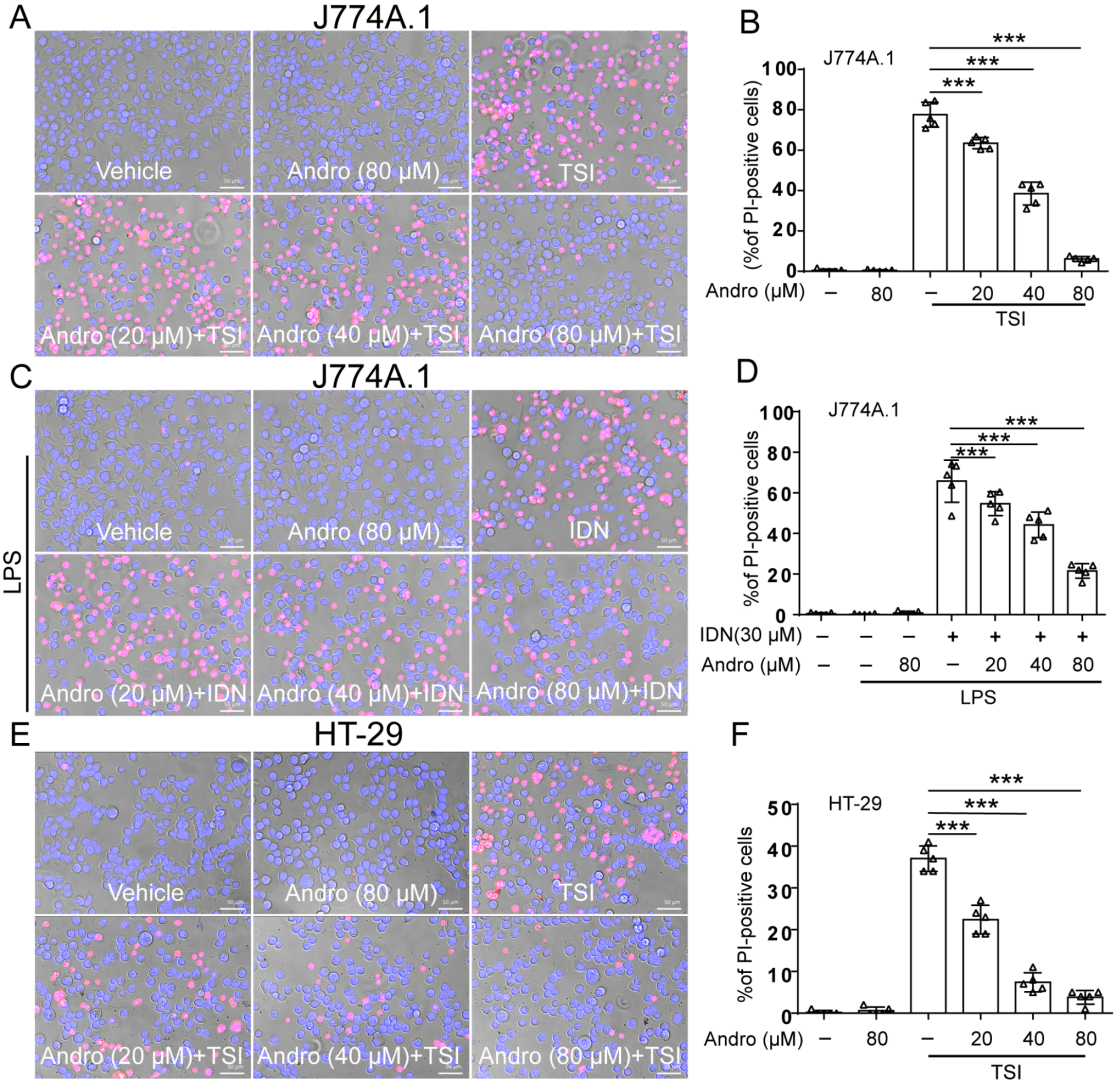
Figure 1 Andro suppresses necroptosis in macrophages and HT-29 cells J774A.1 cells and HT-29 cells were pretreated with or without Andro for 1 h, followed by stimulation with the combination of TNF-α, LCL-161, and IDN-6556 (TSI) (A,B,E,F) or LPS plus IDN-6556 (LI) (C,D) in the presence or absence of Andro for 2 h. The ratios of cell death were evaluated through staining cells with PI (red, cells undergoing lytic cell death) and Hoechst 33342 (blue, all nuclei). Scale bar: 50 μm. Data are shown as the mean ± SD (n = 5). *P < 0.05; ***P < 0.001; ns, not significant.

The anti-necroptotic effect of Andro was evaluated in macrophages and HT-29 cells treated with TSI or LI. Both TSI- and LI-treated cells presented characteristic necroptotic features, including cellular swelling and membrane rupture [24]. As indicated by PI incorporation, Andro dose-dependently inhibited necroptosis in J774A.1 macrophages ( Figure 1A–D), HT-29 adenocarcinoma cells ( Figure 1E,F), and mouse BMDMs ( Supplementary Figure S1A–D). These findings demonstrate that Andro effectively inhibits necroptosis in both macrophages and other cell types.

### Andro suppresses the necroptotic signaling pathway in mouse macrophages

**Figure 2:**
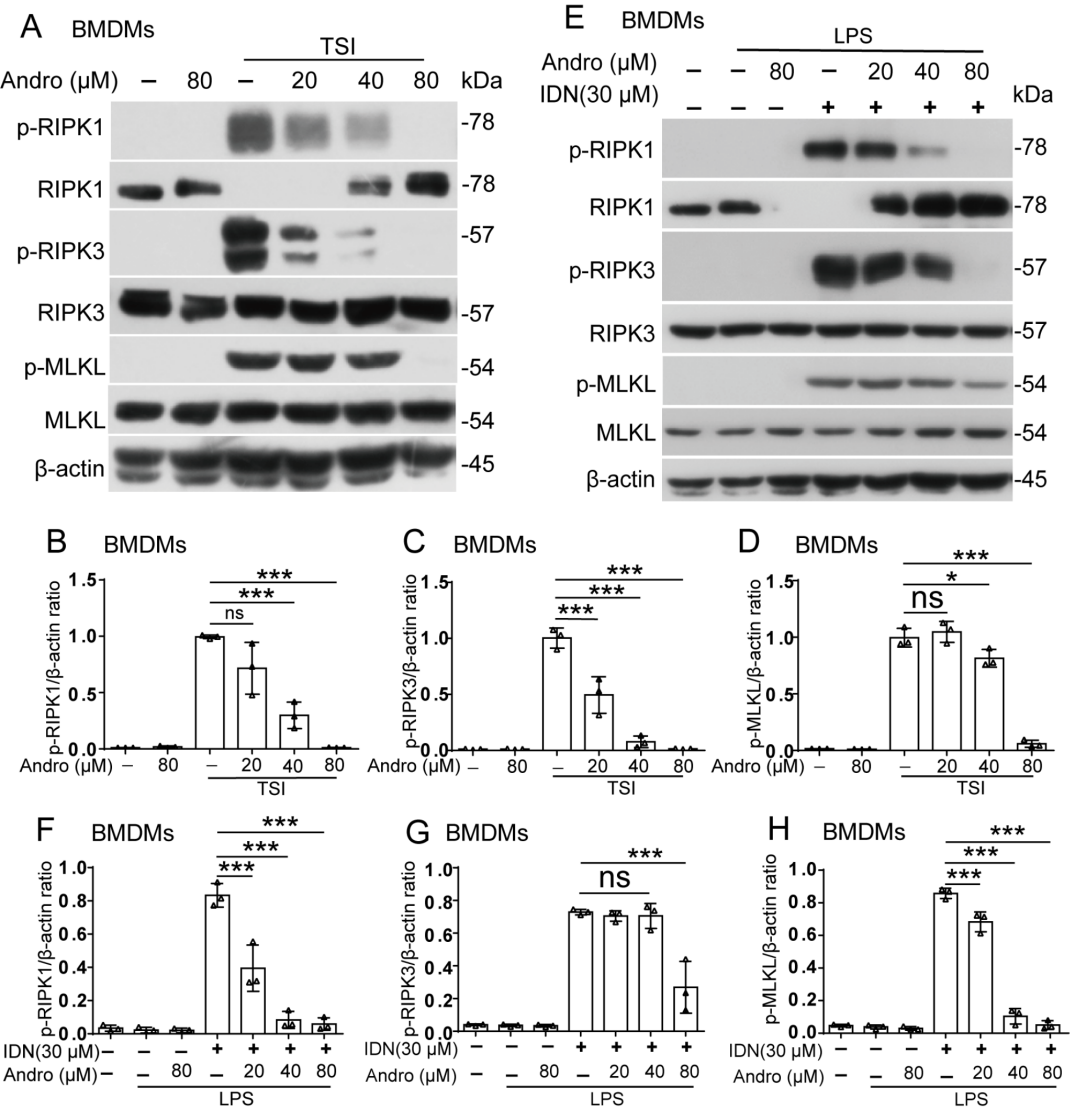
Figure 2 Andro suppresses the necroptotic signaling pathway BMDMs were treated with TNF-α, LCL-161, IDN-6556 (TSI) (A–D), or LPS plus IDN-6556 (LI) (E–H) as described in Figure 1. Western blot analysis was used to detect protein levels involved in the necroptotic pathway. The loading control was β-actin. The levels of the indicated proteins relative to those of β-actin in the cell lysates were determined, with the levels in the TSI and LI groups being set to 1.0. Data are shown as the mean ± SD (n = 3). *P < 0.05; ***P < 0.001; ns, not significant.

Next, we investigated whether Andro inhibits necroptotic signaling pathway activation in TSI- or LI-stimulated macrophages. Western blot analysis revealed that after TSI or LI treatment, the levels of phosphorylated RIPK1, RIPK3, and MLKL were profoundly increased in BMDMs ( Figure 2A–H) and J774A.1 cells ( Supplementary Figure S2A–H), confirming activation of the necroptotic signaling pathway. Andro treatment significantly attenuated these phosphorylation events ( Figure 2 and Supplementary Figure S2), paralleling the inhibition of cell death induced by TSI or LI. These findings indicate that Andro blocks necroptosis by inhibiting the RIPK1/RIPK3/MLKL signaling.

### Andro blocks the formation of RIPK1/RIPK3 necrosomes induced by TSI or LI

**Figure 3:**
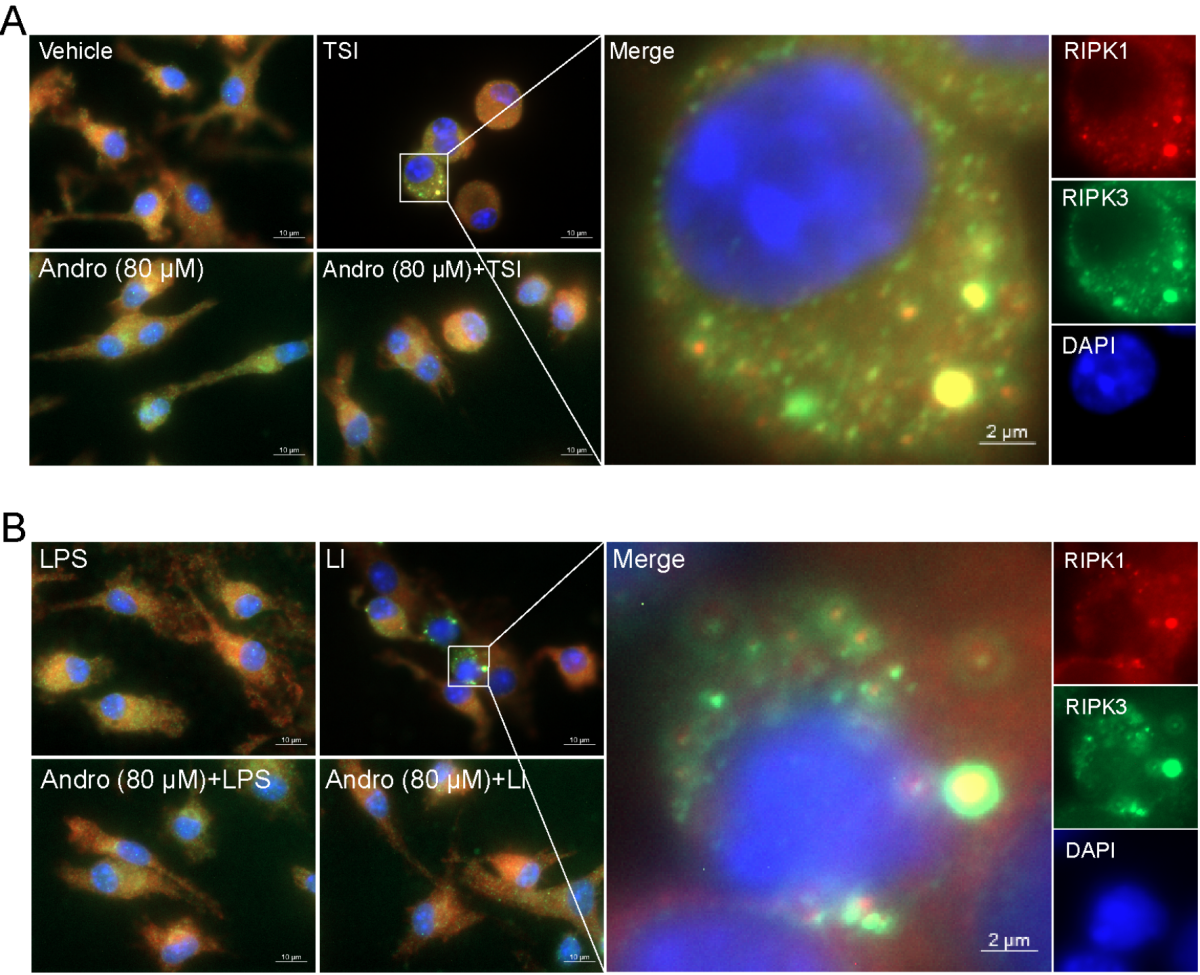
Figure 3 Andro prevents the formation of necrosomes in macrophages stimulated with TSI or LI BMDMs were treated as described in Figure 1. Immunofluorescence was used to observe the distribution and co-localization of RIPK1 (red) and RIPK3 (green). The nuclei were stained with Hoechst 33342 (blue). Scale bar: 10 μm; inset scale bar: 2 μm.

In normal cells, RIPK1 and RIPK3 are diffusely and evenly distributed in the cytoplasm. However, upon necroptotic stimulation, RIPK1 and RIPK3 aggregate into punctate necrosomes in the cytoplasm, with subsequent recruitment and activation of MLKL [18]. In this study, IF analysis was used to investigate whether Andro could inhibit necrosome formation in both TSI-treated ( Figure 3A) and LI-treated ( Figure 3B) BMDMs. RIPK1 (red) or RIPK3 (green) formed aggregates in the cytoplasm, but Andro pretreatment abolished their co-localization. However, such co-localization of RIPK1 and RIPK3 aggregates was not observed in the control, Andro, or Andro + TSI/LI groups. This disruption of necrosome formation by Andro mechanistically explains its anti-necroptotic activity.

### Andro scavenges TSI-induced ROS and prevents ROS-mediated mitochondrial protein oligomerization, thus reducing mitochondrial damage

**Figure 4:**
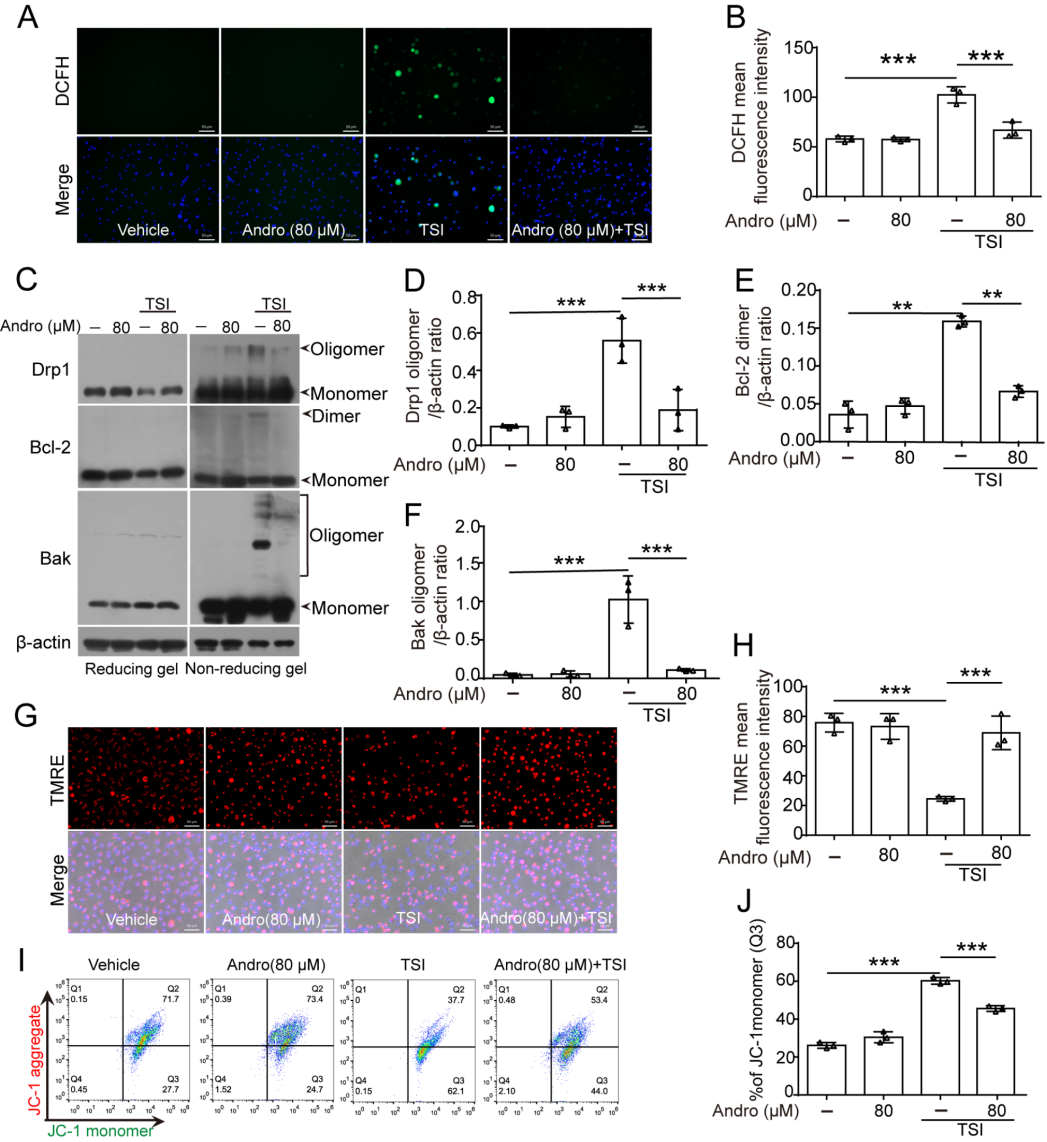
Figure 4 Andro suppresses the production of ROS induced by TSI in BMDMs BMDMs were treated as described in Figure 1. (A) After staining with DCFH-DA, ROS (green) were observed via fluorescence microscopy. (B) Quantitative analysis of DCFH fluorescence intensity in (A). (C) Western blot analysis was performed to examine the expressions of the indicated proteins after reducing and nonreducing protein electrophoresis. (D-F) Relative levels of oligomerized Drp1, Bcl-2, and Bak to that of β-actin in cell lysates were determined, with the levels of the TSI group being set as 1.0. (G) After being stained with TMRE, the cells were observed with a fluorescence microscope. The nuclei were stained with Hoechst 33342 (blue). Scale bar: 50 μm. (H) Mitochondrial membrane potential (MMP) was evaluated as the ratio of TMRE fluorescence-positive cells to control cells. (I) MMP was analyzed by flow cytometry after the cells were stained with JC-1 reagent. (J) Loss of the MMP was calculated as the ratio of cells with JC-1 monomers in (I). Data are shown as the mean ± SD (n = 3). ***P < 0.001.

Reciprocal regulation between ROS and necroptotic signaling has been suggested by emerging evidence: (1) RIPK1 senses ROS via its three key cysteine residues, leading to autophosphorylation at serine 161 and subsequently recruiting RIPK3 to form a functional necrosome [25]; (2) ROS mediate the cross-linking of MLKL proteins through disulfide bonds, facilitating their oligomerization and necroptosis execution [26]; (3) mtROS not only triggers pyroptosis-mediated mtDNA release but also activates the necroptotic signaling pathway [27]; and (4) activated RIPK1 stimulates the generation of ROS, establishing a pathogenic feedforward loop [28]. Therefore, we next sought to investigate whether Andro could suppress the generation of intracellular ROS and mtROS in TSI-stimulated cells. DCFH-DA was used as a probe for intracellular ROS, and MitoSOX was used to detect the mitochondrial superoxide levels in TSI-activated cells. The results showed that Andro significantly suppressed both intracellular ROS ( Figure 4A,B) and mtROS ( Supplementary Figure S3A,B), suggesting that Andro inhibits necroptosis by inhibiting ROS production.

On the basis of our previous findings that both glycometabolic stress and ROS stress induce disulfide-linked oligomerization of certain mitochondrial proteins (Drp1, Bcl-2, and Bak) [21], we examined whether this process takes place under necroptotic conditions. By nonreducing electrophoresis and western blot analysis, oligomerized Drp1, Bcl-2, and Bak were observed in TSI-treated macrophages, which was abolished by Andro treatment ( Figure 4C–F), indicating that ROS-dependent crosslinking of these proteins occurred during necroptosis.

The ROS and oligomerized mitochondrial proteins induced by TSI are likely to cause mitochondrial damage. To substantiate this concept, the reduction in the MMP induced by TSI treatment was detected via both the TMRE assay ( Figure 4G,H) and the JC-1 assay combined with flow cytometry analysis ( Figure 4I,J). TSI caused marked loss of the MMP, as evidenced by decreased TMRE fluorescence ( Figure 4G,H) and increased JC-1 green monomers ( Figure 4I,J), which was reversed by Andro. These data collectively indicate that Andro preserves mitochondrial function by preventing ROS accumulation and subsequent oxidative protein modifications.

### Andro inhibits the incorporation of Bcl-2 and Bak into necrosomes induced by TSI

**Figure 5:**
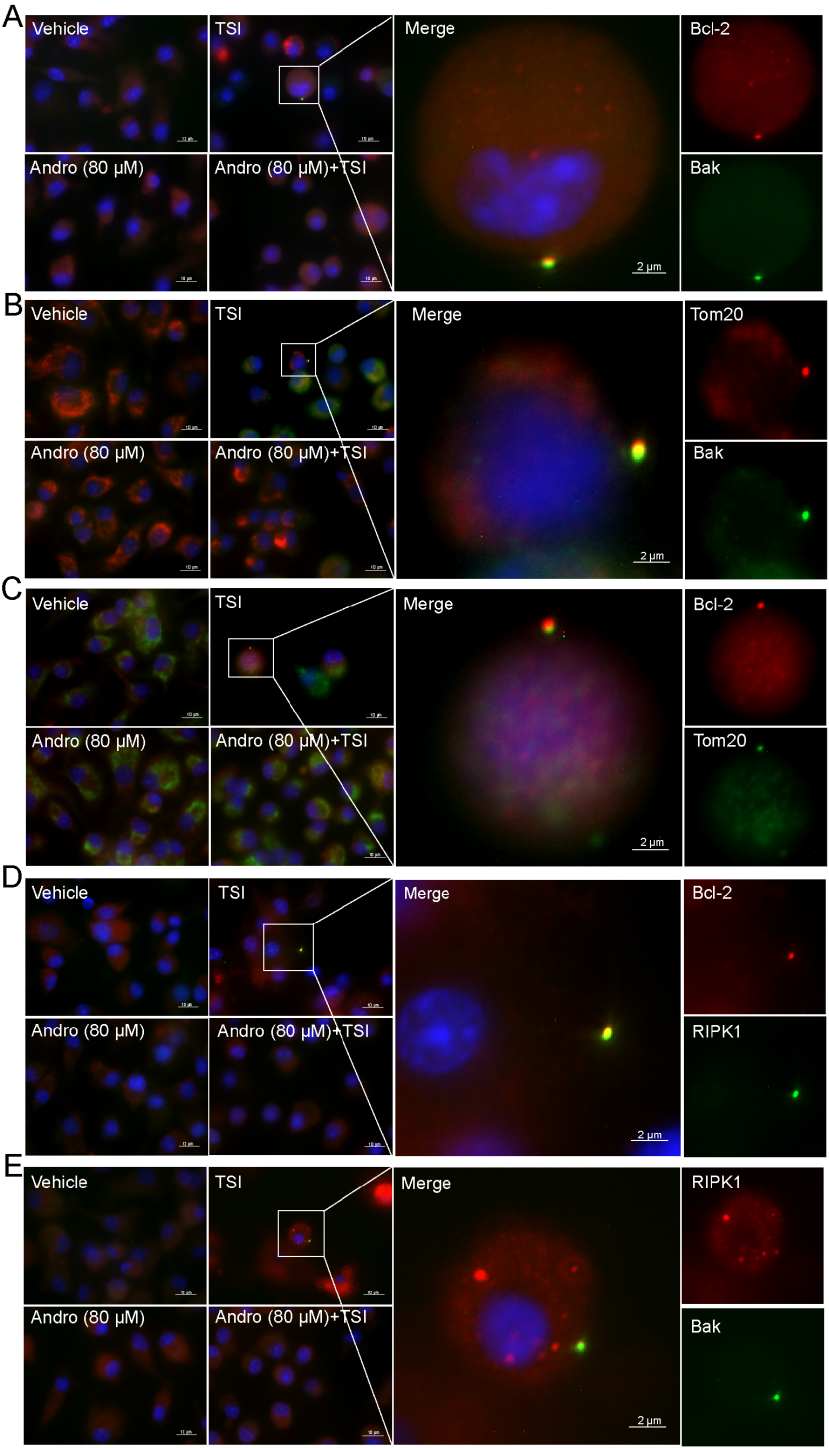
Figure 5 Andro prevents the formation of necrosomes in TSI-induced BMDMs BMDMs were treated with TSI as described in Figure 1. The distribution of the indicated proteins (green or red) was observed via immunofluorescence microscopy. The nuclei were stained with Hoechst 33342 (blue). Scale bar: 10 μm; inset scale bar: 2 μm.

Recent studies have indicated that RIPK1 and the ROS-mediated transfer of MLKL, Bak, and Drp1 to mitochondria lead to mitochondrial damage and necroptosis [29]. We hypothesized that the proteins of the necroptotic pathway might also be transferred to the mitochondria of TSI-treated macrophages in a similar manner to activate necroptosis. The distributions of these proteins were observed via IF microscopy. Our data revealed that Bcl-2, Bak, and Tom20 (a mitochondrial marker) formed a speck in TSI-treated cells ( Figure 5A–C), which co-localized with RIPK1/RIPK3 aggregates ( Figure 5D,E), suggesting the assembly of mitochondria-associated necrosomes. Andro pretreatment abolished these interactions ( Figure 5). The formation of necrosomes comprising Bcl-2, Bak, RIPK1, and RIPK3 likely caused mitochondrial disruption, as the expression of Tom20 was much lower in the TSI group than in other groups ( Figure 5B,C). While our nonreducing gels demonstrated Bcl-2/Bak oligomerization ( Figure 4C), more investigations are warranted to determine whether these proteins in the necrosome have been crosslinked (oligomerized) through disulfide bonds. These findings indicate that ROS-induced mitochondrial protein aggregation may bridge necroptotic signaling and organelle damage.

### Andro induces the activation of the Nrf2-heme oxygenase-1 (HO-1) signaling pathway

**Figure 6:**
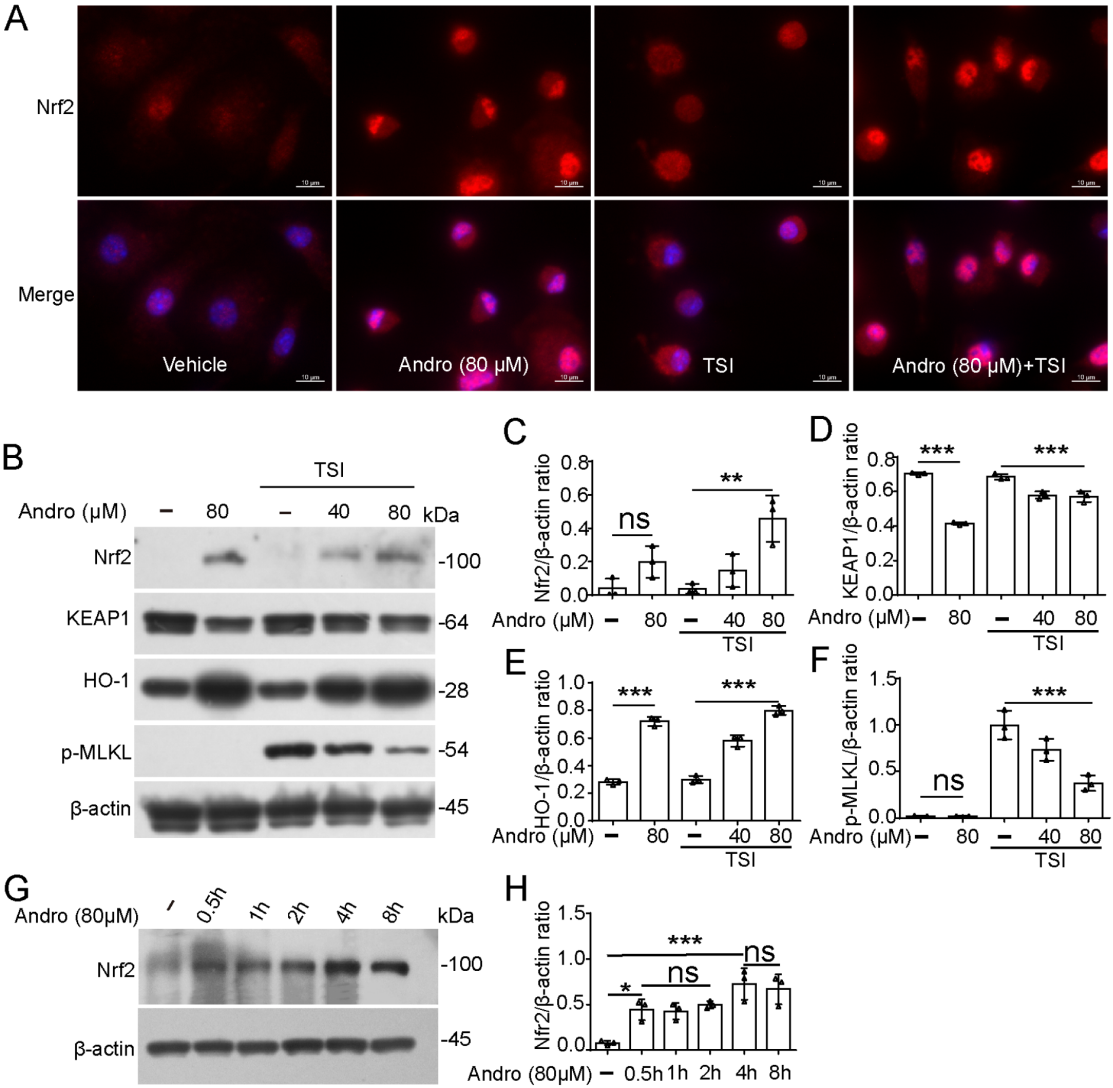
Figure 6 Andro activates the Nrf2 signaling pathway by promoting the translocation of Nrf2 into the nucleus BMDMs were treated as described in Figure 1. (A) The distribution of Nrf2 (red) was observed via immunofluorescence microscopy. The nuclei were stained with Hoechst 33342 (blue). Scale bar: 10 μm. (B) The indicated proteins were analyzed by western blot analysis. β-Actin was used as a loading control. (C–F) The levels of the indicated proteins relative to those of β-actin in the cell lysates were determined, with the levels in the TSI group being set to 1.0. (G,H) BMDMs were treated with Andro for different durations. The levels of Nrf2 in the cell lysates were analyzed by western blot analysis. Data are presented as the mean ± SD (n = 3). *P < 0.05; **P < 0.01; ***P < 0.001; ns, not significant.

HO-1, a cytoprotective enzyme regulated by Nrf2 through antioxidant response elements, plays a critical role in safeguarding cells against oxidative stress [30]. Under normal conditions, Nrf2 is maintained at a low level, as it is sequestered in the cytoplasm by binding to its cytoplasmic inhibitor KEAP1 and tends to be degraded through the ubiquitin-proteasome pathway. Oxidative stress disrupts this interaction, enabling Nrf2 to translocate to the nucleus, where it transcriptionally upregulates antioxidant-related proteins, including HO-1 [30]. Given that Andro inhibits pyroptosis by promoting the expression of Nrf2 [31], we investigated whether the inhibitory effect of Andro on necroptosis depends on its regulation of Nrf2 expression. IF microscopy revealed that the translocation of Nrf2 to the nucleus was significantly increased after Andro treatment, irrespective of TSI treatment ( Figure 6A). Consistent with this observation, western blot analysis confirmed that Nrf2 levels were undetectable or minimal in the control and TSI-only groups but were greatly increased in Andro-treated cells, regardless of TSI treatment ( Figure 6B,C). Consistent with the findings of Nrf2 activation, KEAP1 levels were reduced, whereas HO-1 levels were elevated by Andro ( Figure 6B,D,E). Thus, Andro alone was able to induce the activation of the Nrf2-HO-1 signaling pathway. Indeed, Andro increased the levels of Nrf2 in a time-dependent manner, with rapid Nrf2 induction occurring within 0.5 h of Andro exposure ( Figure 6G,H). Importantly, the levels of Nrf2 and HO-1 inversely correlated with that of p-MLKL in TSI-treated cells ( Figure 6B,F), suggesting that Andro inhibits necroptosis by activating the Nrf2-HO-1 signaling pathway.

### Derivatives of Andro without Nrf2 activation capacity do not have anti-necroptotic effects on necroptotic inducers

**Figure 7:**
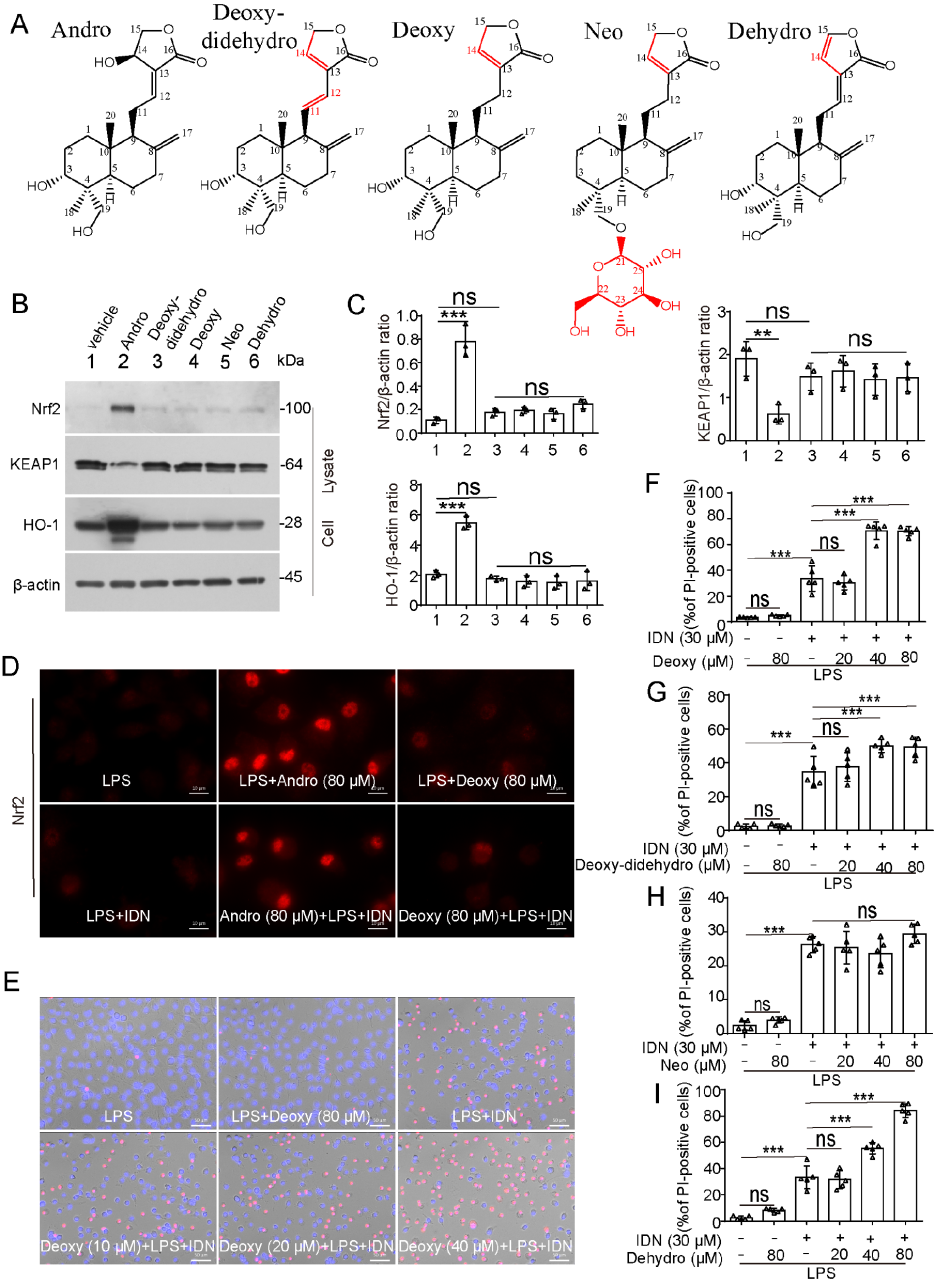
Figure 7 The anti-necroptotic activity of Andro, rather than its derivatives, is related to its activation of the Nrf2-HO-1 signaling pathway (A) Chemical structure formulas of andrographolide (Andro), dehydroandrographolide (Dehydro), neoandrographolide (Neo), 14-deoxy-11,12-didehydroandrographolide (Deoxy-didehydro), and 14-deoxyandrographolide (Deoxy). (B,C) BMDMs were treated with Andro or its derivatives for 8 h. Protein expression levels were analyzed by western blot analysis. (D–I) BMDMs were pretreated with or without Andro or its derivatives for 1 h, followed by stimulation with LPS plus IDN-6556 in the presence or absence of Andro or its derivatives for 2 h. (D) The distribution of Nrf2 (red) was observed via immunofluorescence microscopy. The nuclei were stained with Hoechst 33342 (blue). Scale bar: 10 μm. (E) Cell death was evaluated by staining cells with PI (red) and Hoechst 33342 (blue). Scale bar: 50 μm. (F–I) The proportions of PI-positive cells relative to the total number of cells stained with Hoechst 33342 were calculated. Data are shown as the mean ± SD (n = 5). **P < 0.01; ***P < 0.001; ns, not significant.

To establish structure‒activity relationships, we compared Andro with its derivatives, including dehydroandrographolide (Dehydro), neoandrographolide (Neo), 14-deoxy-11,12-didehydroandrographolide (Deoxy-didehydro), and 14-deoxyandrographolide (Deoxy) ( Figure 7A). Andro is a labdane diterpenoid with three hydroxyl groups (-OH) at positions C-3, -14, and -19 and a double bond at C-12(13). Compared with Andro, Deoxy and Dehydro have lost the C-12(13) double bond, whereas Deoxy-didehydro shifts it to the position C-11(12); Neo links a glucoside moiety to its C-19 hydroxyl group (which improves water solubility) but loses the C-3 hydroxyl group; and all four derivatives have lost the C-14 hydroxyl group. Among these chemicals, only parent Andro profoundly activated Nrf2-HO-1 signaling ( Figure 7B,C). Compared with 14-deoxyandro–treated cells, Andro-treated cells presented substantially stronger nuclear Nrf2 immunofluorescence ( Figure 7D). Functional assays revealed that none of the derivatives inhibited the cell death induced by LI; conversely, Deoxy, Deoxy-didehydro, and Dehydro even dose-dependently exacerbated death ( Figure 7E–I). These results suggest that Nrf2-HO-1 activation is essential for the anti-necroptotic activity of Andro.

### Andro alleviates uterus injury induced by TNF-α in mice

**Figure 8:**
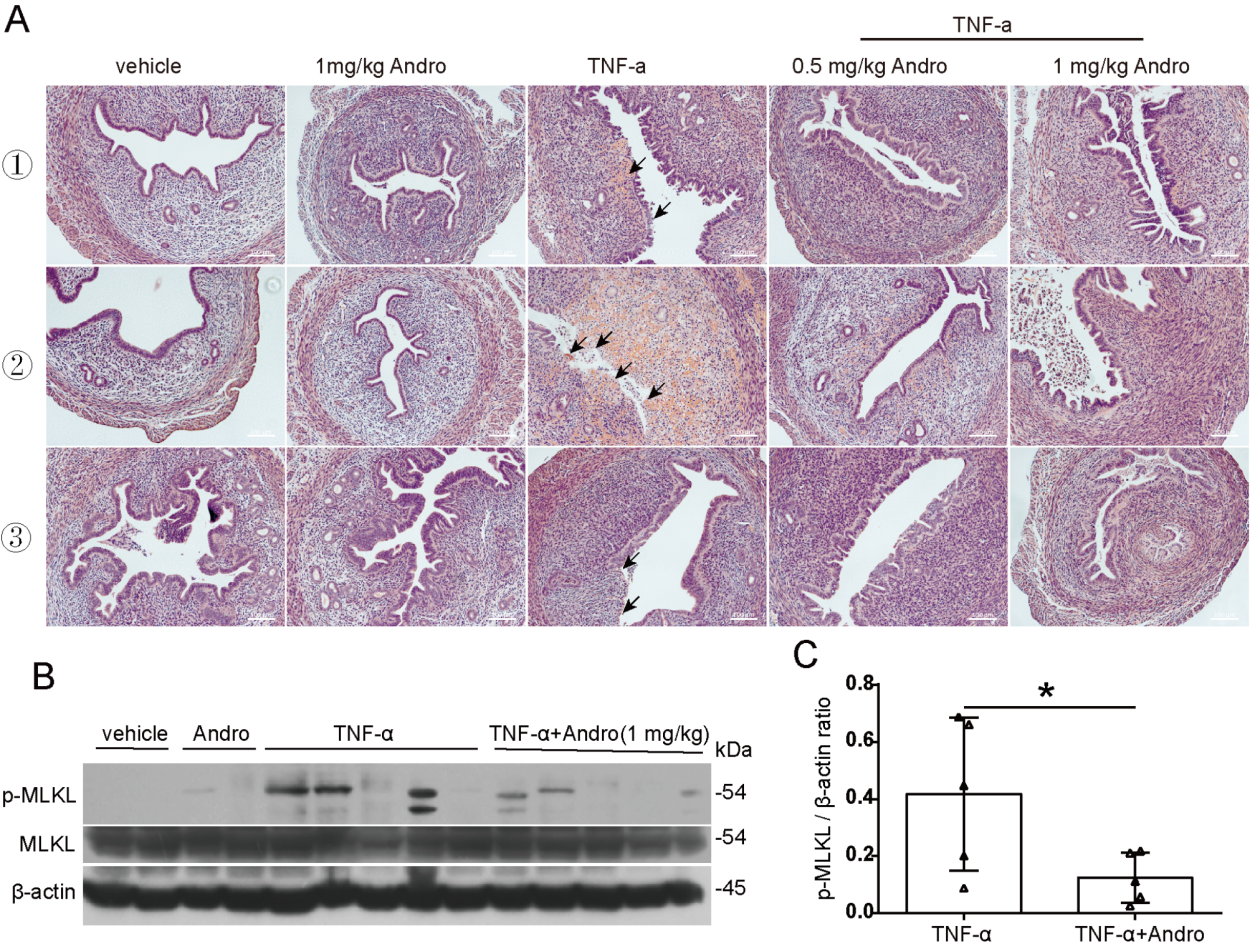
Figure 8 Andro mitigates the severity of TNF-α-induced uterus injury in mice Wild-type female mice were intraperitoneally injected with sterile PBS or Andro three times, 1 h before, 2 h after, or 5 h after the injection of TNF-α (5 μg per mouse) via the tail vein. The uteri were collected 9 h after challenge with TNF-α. (A) Representative H&E-stained histology of uteri from the mice. Microscopy images of the histological sections are shown at 100× magnification. Scale bar: 100 μm. Epithelial injury is indicated by black arrows. (B) Western blot analysis of p-MLKL expression in uterus tissues from each group. The loading control was β-actin. (C) The levels of p-MLKL relative to those of β-actin were determined. An unpaired Student’s t test was used to evaluate the differences between the two groups. Data are shown as the mean ± SD (n = 5). *P < 0.05.

Given the established role of TNF-α in inducing necroptosis-driven inflammation [18], we evaluated the anti-inflammatory effect of Andro on TNF-α-induced systemic inflammatory response syndrome (SIRS) in mice. Histopathological analysis revealed extensive uterine necrosis in the TNF-α-treated group ( Figure 8A). With Andro treatment, however, the uterus injury induced by TNF-α was markedly alleviated ( Figure 8A). Western blot analysis revealed that Andro reduced the level of p-MLKL ( Figure 8B,C), confirming the suppression of necroptosis. These results show that Andro at low doses (< 1 mg/kg, bw) can alleviate inflammation associated with necroptosis.

## Discussion

Previous studies have established the dual suppressive effects of Andro on apoptosis [32] and pyroptosis [ 10, 33], but its potential modulatory role in necroptosis remains unknown. The findings of the current study demonstrate the broad-spectrum inhibitory capacity of Andro against LI- or TSI-induced necroptosis in macrophages and other cell types. Further investigation of the underlying mechanisms revealed that Andro blocks the RIPK1/RIPK3/MLKL pathway while upregulating Nrf2 and its downstream antioxidant targets, thus decreasing intracellular ROS and preserving the MMP. These results suggest that Andro can be used as an effective inhibitor of necroptosis.

Necroptosis contributes to diverse pathological conditions, such as kidney injury [34], heart diseases [35], cancers [36], and neurodegenerative diseases [37]. Several inhibitors have been developed to target the RIPK1-RIPK3-MLKL necroptotic pathway, including Nec-1 (targeting RIPK1) and GSK′872 (targeting RIPK3) [38]. Notably, ROS-mediated cysteine oxidation in RIPK1 initiates autophosphorylation; subsequently, p-RIPK1 recruits RIPK3 to form a necrosome, ultimately leading to necroptosis [ 25, 39]. However, the precise mechanism, especially how ROS regulate the RIPK1-RIPK3-MLKL pathway, remains unclear. Our group’s prior studies revealed that mtROS scavenging via the inhibition of reverse electron transport prevents necroptosis and PANoptosis [ 18, 40]. Recently, our laboratory reported that some phytochemicals, including baicalin [24] and celastrol [22], can inhibit necroptosis by suppressing mtROS. Consistent with this finding, Andro effectively attenuated both total ROS and mtROS while preserving mitochondrial integrity.

Previously, Andro was reported to target Drp1, a protein that is fundamental for mitochondrial and peroxisomal fission [41]. Given that the RIPK1/ROS-mediated translocation of MLKL, Bak, and Drp1 to the mitochondria drives MMP collapse and ultimately results in necroptosis [29], Andro’s inhibition of Drp1, Bcl-2, and Bak oligomerization suggests possible cross-talk between the mitochondrial fission machinery and necrosome formation; however, whether it inhibits necroptosis by directly targeting Drp1 needs further investigation.

Another reported target of Andro is KEAP1 [42], which is a key regulator of cellular redox homeostasis, as described above [43]. The KEAP1/Nrf2 regulatory network extends to the thioredoxin (Trx) and glutathione (GSH) systems, which coordinately maintain the native thiol status of proteins [44]. In this study, we showed that the protein levels of Nrf2 and HO-1 were increased upon Andro treatment both in the presence and absence of necroptotic inducers (TSIs). Interestingly, the levels of Nrf2 and HO-1 were inversely correlated with the level of p-MLKL, a marker of necroptosis. The inability of derivatives to activate Nrf2-HO-1 signaling or inhibit necroptosis further supports the essential role of this pathway.

While our study established Nrf2-HO-1 activation as a key antinecroptotic mechanism, it is unclear how Nrf2 and HO-1 precisely regulate the formation of necrosomes. Since the upregulation of Nrf2 and HO-1 by Andro has been reported in various studies [ 45 - 47], we hypothesize that Andro likely targets KEAP1 to prevent ROS bursts. Indeed, the results of the present study revealed that Andro prevented the oligomerization of several mitochondrial proteins that are critical for cell survival, such as Bcl-2 and Bak, through disulfide bonds. These cross-linked proteins not only induce mitochondrial damage but also participate in necrosome assembly, suggesting a feedforward mechanism whereby oxidized Bcl-2/Bak both induce mitochondrial permeabilization and serve as necrosome scaffolds. Validating this hypothesis requires advanced techniques such as *in situ* crosslinking proteomics and real-time ROS biosensor imaging.

Our study also revealed that Andro at low doses (≤ 1 mg/kg, bw) could alleviate the uterus damage induced by TNF-α in mice. Consistent with our previous SIRS findings [18], TNF-α induced necroptosis in uterus tissues, which was significantly suppressed by Andro treatment. Notably, Andro consistently upregulated Nrf2 and HO-1 levels irrespective of TSI co-stimulation. Although our study revealed that short-term and low-dose Andro treatment has no cytotoxic effect on normal cells, caution is warranted since chronic/high-dose toxicity has been highlighted in comprehensive Andro pharmacological reviews [48]. Nonetheless, given its remarkable anti-necroptotic and anti-inflammatory properties, Andro can potentially be developed as an external medicine [48].

In this study, we showed that Andro could inhibit necroptosis, but its derivatives failed, indicating critical structure‒activity relationships. All 4 derivatives and Andro have been reported to have anti-inflammatory and anticancer properties [ 49– 51], as well as protective effects on hepatotoxicity induced by toxins [ 52– 55]. They also have viricidal activities [56], but the spectrum of viruses they can kill varies greatly. For example, Andro and Deoxy can kill foot-and-mouth disease virus 3C ^pro^, but Neo cannot [57]. Similarly, Andro and Deoxy-didehydro significantly inhibited thrombin-induced platelet aggregation, but Neo exhibited little or no activity [58]. The targeting and bioavailability of the derivatives and Andro differ because of their slight structural differences, such as differences in double bonds and hydroxyl substitutions ( Figure 7A). Systematic comparisons of derivative pharmacodynamics could illuminate optimal molecular scaffolds for pathway-specific drug design.

In summary, the results of this study suggest that Andro can inhibit necroptosis by reducing ROS, protecting mitochondria from MMP loss, and inhibiting the formation of necrosomes. However, the inability of derivatives to activate Nrf2/HO-1 signaling or inhibit necroptosis highlights Andro’s unique structural requirements. Our data suggest that Andro is a potential phytochemical for treating necroptosis-related diseases.

## Supporting information

25010supplementary_figures

## Supplementary Data

Supplementary data is available at *Acta Biochimica et Biophysica Sinica* online.
