# Supplementary material for: Andrographolide prevents necroptosis by suppressing the generation of reactive oxygen species: Andro prevents necroptosis by suppressing the generation of ROS
Source: Acta Biochim Biophys Sin (Shanghai). 2025 May 28;57(12):2048–61. doi: 10.3724/abbs.2025077 (PMC12747933; doi:10.3724/abbs.2025077)
Supplement: 25010supplementary_figures [file 25010supplementary_figures.docx]

**
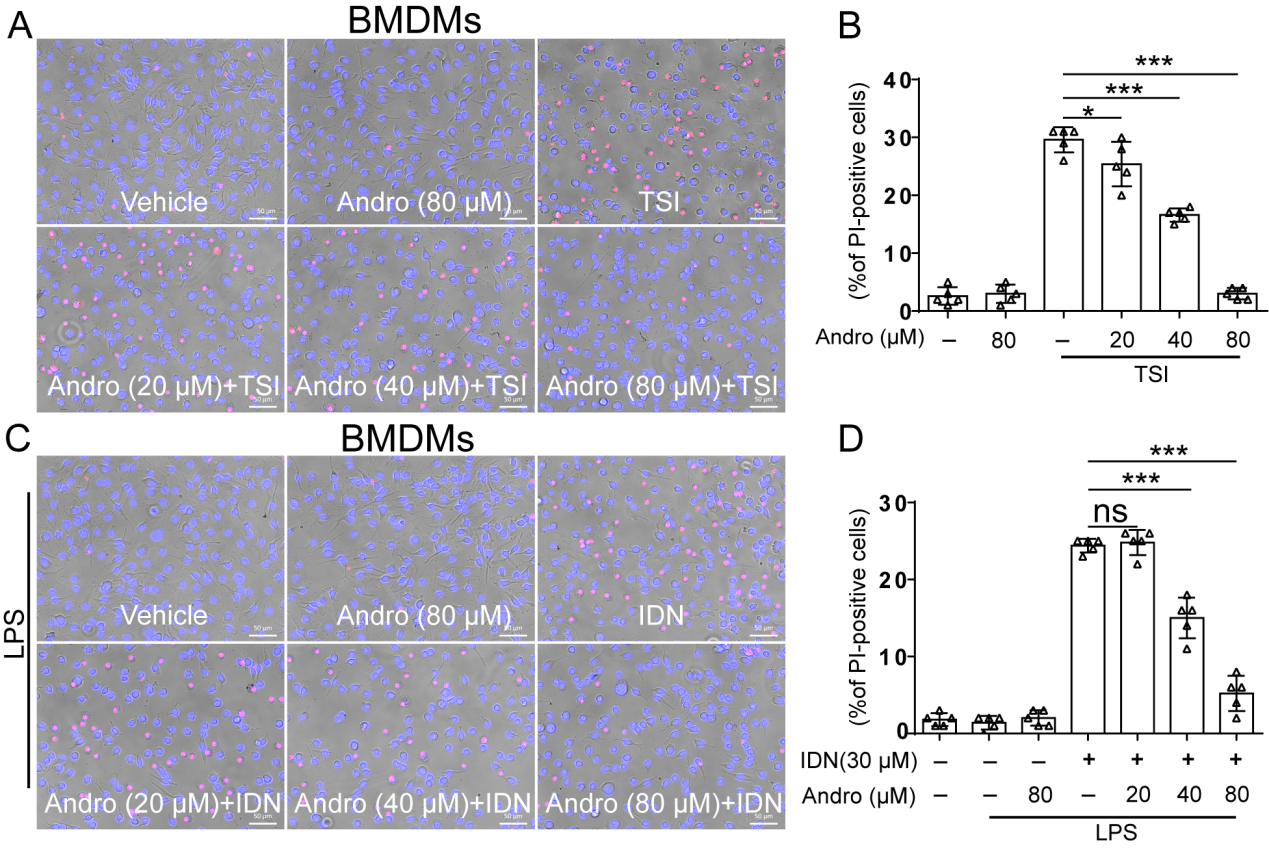
**

**Supplementary Figure S1. Andro suppresses necroptosis in macrophages**  Bone marrow-derived macrophages (BMDMs) were pretreated with or without Andro for 1 h, followed by stimulation with the combination of TNF-α, LCL-161, and IDN-6556 (TSI) (A,B), or LPS plus IDN-6556 (LI) (C,D) in the presence or absence of Andro for 2 h. Cell death was evaluated by staining cells with PI (red, staining lytic cells) and Hoechst 33342 (blue, staining all nuclei). Scale bar: 50 μm. Data are shown as the mean ± SD (*n* = 5). **P* < 0.05; ****P* < 0.001; ns, not significant.

**
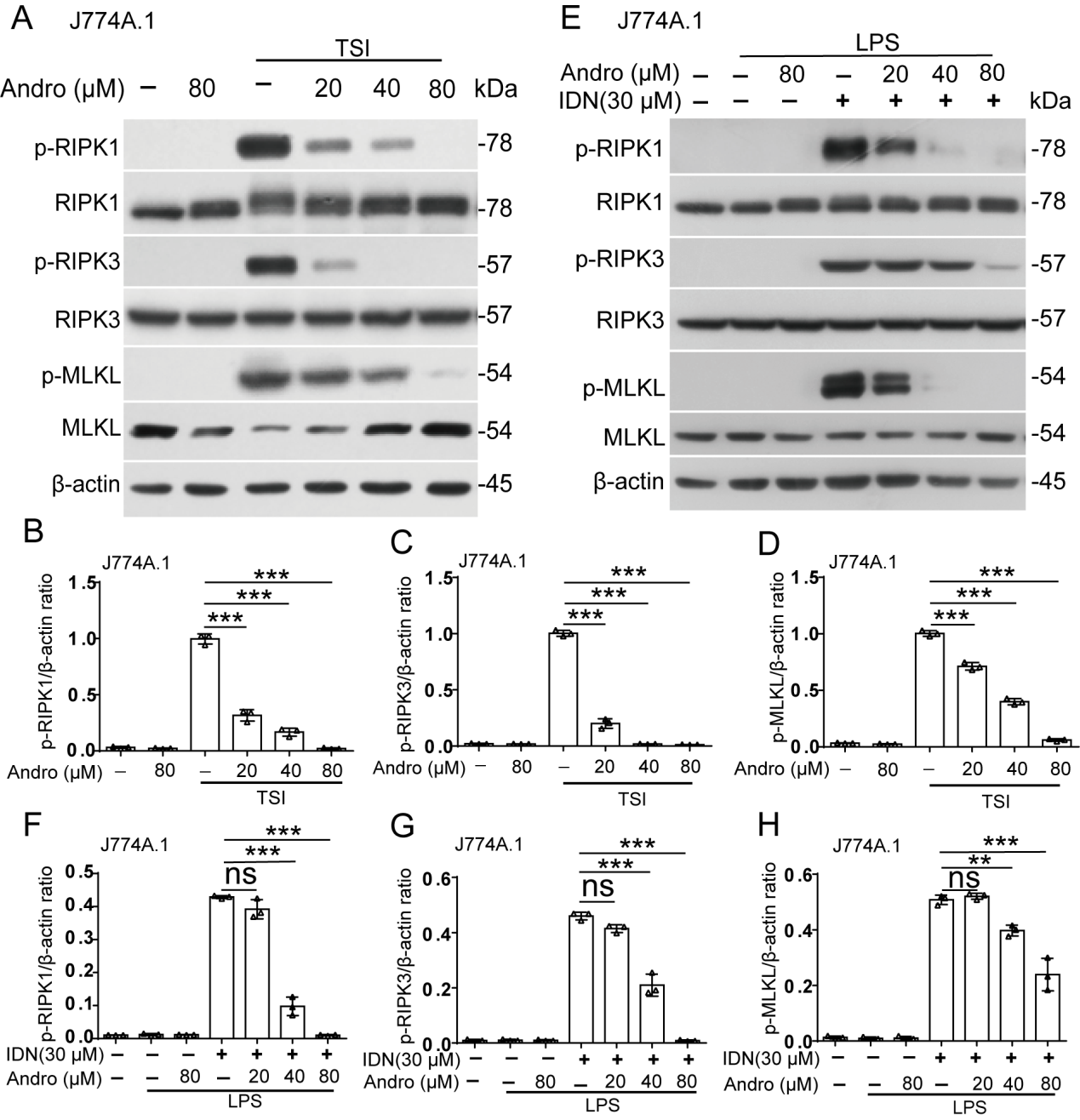
**

**Supplementary Figure S2. Andrographolide suppresses the necroptotic signaling pathway** J774A.1 cells were treated with TNF-α, LCL-161, and IDN-6556 (TSI) (A−D), or LPS plus IDN-6556 (LI) (E−H) as described in **Figure. 1**. Western blot analysis was used to detect the proteins of the necroptotic pathway. The loading control was β-actin. Relative levels of indicated proteins to that of β-actin in cell lysates were determined, with the levels of the TSI or LI group being set as 1.0, respectively. Data are shown as the mean ± SD (*n* = 3). ***P* < 0.01; ****P* < 0.001; ns, not significant.

**
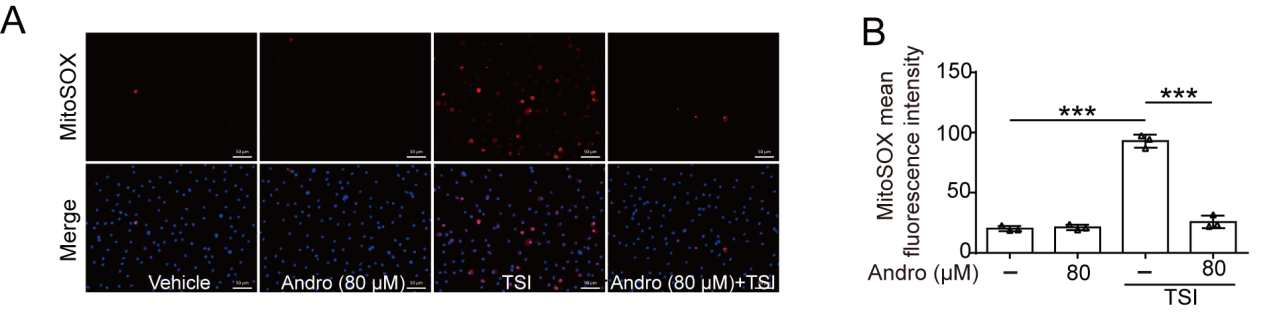
**

**Supplementary Figure S3. Andrographolide reduces mitochondrial reactive oxygen species (ROS) in TSI-treated macrophages** (A) Bone marrow-derived macrophages (BMDMs) were treated as described in **Figure. 1**. After staining the cells with mitoSOX, mitochondrial superoxides (red) were observed by fluorescence microscopy. The nuclei were stained with Hoechst 33342 (blue). (B) Ratios of cells with mitochondrial superoxides to all cells. ****P* < 0.001.
